# Detection of Post-COVID-19 Patients Using Medical Scent Detection Dogs—A Pilot Study

**DOI:** 10.3389/fmed.2022.877259

**Published:** 2022-06-16

**Authors:** Friederike Twele, Nele Alexandra ten Hagen, Sebastian Meller, Claudia Schulz, Albert Osterhaus, Paula Jendrny, Hans Ebbers, Isabell Pink, Nora Drick, Tobias Welte, Esther Schalke, Holger Andreas Volk

**Affiliations:** ^1^Department of Small Animal Medicine and Surgery, University of Veterinary Medicine Hannover, Hannover, Germany; ^2^Research Center for Emerging Infections and Zoonoses, University of Veterinary Medicine Hannover, Hannover, Germany; ^3^KynoScience UG, Hörstel, Germany; ^4^Department of Respiratory Medicine, Hannover Medical School, Hannover, Germany; ^5^Bundeswehr Medical Service Headquarters, Koblenz, Germany; ^6^Center for Systems Neuroscience, Hannover, Germany

**Keywords:** SARS-CoV-2, scent detection dogs, Long COVID, volatile organic compound (VOC), COVID-19

## Abstract

There is a growing number of COVID-19 patients experiencing long-term symptoms months after their acute SARS-CoV-2 infection. Previous research proved dogs' ability to detect acute SARS-CoV-2 infections, but has not yet shown if dogs also indicate samples of patients with post-COVID-19 condition (Long COVID). Nine dogs, previously trained to detect samples of acute COVID-19 patients, were confronted with samples of Long COVID patients in two testing scenarios. In test scenario I (samples of acute COVID-19 vs. Long COVID) dogs achieved a mean sensitivity (for acute COVID-19) of 86.7% (95%CI: 75.4–98.0%) and a specificity of 95.8% (95%CI: 92.5–99.0%). When dogs were confronted with Long COVID and negative control samples in scenario IIa, dogs achieved a mean sensitivity (for Long COVID) of 94.4 (95%CI: 70.5–100.0%) and a specificity of 96.1% (95%CI: 87.6–100.0%). In comparison, when acute SARS-CoV-2 positive samples and negative control samples were comparatively presented (scenario IIb), a mean sensitivity of 86.9 (95%CI: 55.7–100.0%) and a specificity of 88.1% (95%CI: 82.7–93.6%) was attained. This pilot study supports the hypothesis of volatile organic compounds (VOCs) being long-term present after the initial infection in post-COVID-19 patients. Detection dogs, trained with samples of acute COVID-19 patients, also identified samples of Long COVID patients with a high sensitivity when presented next to samples of healthy individuals. This data may be used for further studies evaluating the pathophysiology underlying Long COVID and the composition of specific VOC-patterns released by SARS-CoV-2 infected patients throughout the course of this complex disease.

## Introduction

Due to their extraordinary olfaction capabilities and trainability dogs can be deployed not only for the detection of explosives, drugs, or missing persons but also for the identification of medical conditions including viral infections (1, 2). Since April 2020, we have been training and deploying dogs to detect samples from individuals with severe acute respiratory syndrome coronavirus 2 (SARS-CoV-2) infection, using different human body fluids, such as sweat, saliva and urine of infected patients (3, 4). With samples of acute coronavirus disease 2019 (COVID-19)-patients, several research groups have shown a detection sensitivity close to 95% and a specificity of 97% for confirmed cases (positive RT-PCR) vs. SARS-CoV-2-negative subjects (3–8). In addition, our group has shown that dogs can differentiate SARS-Co-V-2 infected material not only from control samples but also from samples of patients with other respiratory viral infections, including other coronaviruses (9). It is thought that the specific odor of an infection is composed of a unique pattern of volatile organic compounds (VOCs). The specific VOC pattern of a SARS-CoV-2 infected individual as well as of those with other viral infections is still under investigation (10). It is interesting to note that canines, in contrast to humans, have the Jacobson vomero-nasal organ (VNO), which is characterized by a different mechanism of odor perception, of which the main function is intra-species communication *via* the detection of pheromones, but it can also sense a wide variety of molecules (11). It has been speculated that VNO may detect viral proteins (11).

Whereas, most patients fully recover from COVID-19, a significant proportion experiences long-term-symptoms (12). A recent study found a prevalence of post-acute symptoms among people with COVID-19 in the UK between 3.0% (based on tracking specific symptoms) to 11.7% (based on self-classification) (13). The WHO recently published a clinical case definition of post-COVID-19 condition by a Delphi consensus (12). According to the WHO “post-COVID-19 condition occurs in individuals with a history of SARS-CoV-2 infection, usually 3 months from onset of COVID-19 with symptoms that last for at least 12 months and cannot be explained by an alternative diagnosis” (12). Common symptoms include fatigue, shortness of breath, muscle pain, cough, cognitive impairment, memory loss and sleep disorders, all leading to reduced quality of life of affected patients (14). In the following we will refer to post-COVID-19 condition as “Long COVID.”

Up to now, the underlying mechanisms of Long COVID are not fully understood, and current studies are now gradually providing valid data to better understand this condition. One widely discussed hypothesis for the underlying cause of Long COVID is the persistence of viral RNA. The persistence of SARS-CoV-2-RNA has been described for olfactory slots (15), brain (16), whereas viral protein persistence has been detected in monocytes (17).

Therefore, it is of great scientific interest to assess whether COVID-19-detection-dogs, trained with samples of acutely SARS-CoV-2-infected patients, can identify samples of Long COVID patients as SARS-CoV-2-positive, as this would support the hypothesis of SARS-CoV-2-persistence or persistent metabolic alterations leading to characteristic VOC patterns in Long COVID patients.

## Materials and Methods

Long COVID patients were recruited at the Department of Respiratory Medicine at Hannover Medical School (MHH; ethic consent number 9042_BO_K_2020). All patients had an initial acute infection with SARS-CoV-2 (verified by RT-qPCR) and prolonged symptoms. Saliva samples (1–3 ml) were collected at the MHH and immediately deep-frozen at −80°C in the laboratory until usage. In addition to saliva samples of Long COVID-patients, negative saliva, urine and sweat from healthy individuals (SARS-CoV-2 RT-qPCR negative, with no previous history of COVID-19, nor a history of a recent cold or infection, recruited at multiple locations) as well as saliva, urine and sweat samples of acute COVID-19 patients (SARS-CoV-2-RT-qPCR positive, hospitalized as well as non-hospitalized) were included in the study as described in detail by Jendrny et al. (3, 4). Based on former results showing that beta-propiolactone (BPL) inactivation does not change scent dog detection, all samples of acute COVID-19 patients and Long COVID patients were inactivated with BPL according to the protocol described in Jendrny et al. to provide safe training conditions for dogs and handlers (3, 4). Characteristics of the recruited patients are summarized in Supplementary Table 1. A volume of 100 μl per sample was pipetted onto a cotton swab (for saliva and urine) or the cotton pad that was used to acquire the sweat sample itself was placed into a 4 ml glass tube and placed in a device called “Detection Dog Training System” (DDTS, Kynoscience UG, Hörstel, Germany) for training and testing as described in our previous studies (3, 4, 9). The DDTS allows for rapid, automatic, randomized, trainer-bias devoid and double-blind sample presentation (3, 4, 9). To verify the recorded results of the DDTS the dogs were filmed during testing and the videos were analyzed manually. In total, nine dogs (seven females and two males) were included. All dogs completed obedience training before the study, were all trained for detection of acute SARS-CoV-2-positive samples and participated in our former studies (3, 4, 9). For the present study the training period could be shortened to 3 days as all dogs were still able to distinguish positive and negative samples with high accuracies. We used saliva, urine and sweat samples of SARS-CoV-2-RT-qPCR positive patients (inactivated with BPL) and of SARS-CoV-2 RT-qPCR negative individuals for training. Samples used for training were never presented again to the dogs during the subsequent testing procedure, guaranteeing novelty of samples for validation purpose.

Two test scenarios were performed. For test scenario I, acute SARS-CoV-2 positive saliva samples and Long COVID saliva samples were presented to the dogs *via* DDTS. In test scenario II, acute SARS-CoV-2 positive (saliva, sweat and urine), Long COVID (saliva) as well as SARS-CoV-2-negative control samples (saliva, sweat and urine) samples were placed in the DDTS. In test scenario II, either dogs were confronted with a Long COVID sample next to SARS-CoV-2-negative control samples (test scenario IIa) or an acute SARS-CoV-2-positive sample was presented next to SARS-CoV-2-negative control samples (test scenario IIb).

Every nose dip into the DDTS' slots was evaluated with four possible options as described before (3, 4, 9). The DDTS changed the positions of the presented samples without letting the dog, dog handler nor other personnel present in the testing room know the new positions of negative or positive samples. This allowed a double-blind sample presentation, controlled and recorded only by the DDTS' software and additional confirmation videos. In addition, all staff involved was positioned accordingly to prevent any interaction or influencing of the animals during the study.

Sample size and sample acquisition were conducted based on and according to our former studies (3, 4, 9). The diagnostic sensitivity as well as diagnostic specificity, positive predictive values (PPV), and negative predictive values (NPV) were calculated according to Trevethan (18). Ninety-five percent confidence intervals (CIs) for sensitivity, specificity, PPV, and NPV were calculated with the hybrid Wilson/Brown method (19). Means of sensitivity, specificity, PPV, NPV, and accuracy with corresponding 95% CIs of mean were also calculated per session. Two-tailed Fisher's exact test was used for analysis of the individual contingency tables; a *P* ≤ 0.05 was considered significant. All calculations were done with the Prism 9 software from GraphPad (La Jolla, CA, USA).

The study was carried out in accordance with the ethical requirements established by the Declaration of Helsinki and was approved by the local Ethics Committee of MHH (ethic consent number 9042_BO_K_2020). Written informed consent from all participants was obtained before sample collection. Animal work according to the study protocol and design was approved by the German Armed Forces.

## Results

Overall, a total of 732 sample presentations were performed (Tables 1–3). When presenting acute COVID-19 samples and Long COVID samples (test scenario I), dogs made 436 rejections and only 18 indications of Long COVID samples (96.04 vs. 3.96%), while 77 correct indications and only 13 false rejections of acute COVID-19 samples were recorded. When presenting Long COVID samples next to SARS-CoV-2 negative samples (test scenario IIa), dogs only rejected a Long COVID sample once, while they indicated 13 Long COVID samples (7.14 vs. 92.86%). During this sample presentation in test scenario IIa, 47 correct rejections and only 2 false indications of SARS-CoV-2-negative samples were performed. During the presentation of acute COVID-19 vs. SARS-CoV-2-negative samples (test scenario IIb), 16 correct indications and 3 false rejections of acute COVID-19 samples were recorded, while 93 correct rejections and only 13 false indications of SARS-CoV-2 negative samples were made.

**Table 1:** Diagnostic performance of the scent detection dogs in test scenario I (Acute COVID-19 vs. Long COVID).

**Dog**	**Detection**	**SARS-CoV-2 disease status**		**Total number of presented samples**	**Diagnostic specificity (Sp)**	**Diagnostic sensitivity (Se)**	**Confidence interval (95% CI) Sp**	**Confidence interval (95% CI) Se**	**Positive predictive value (PPV)**	**Negative predictive value (NPV)**	**Confidence interval (95% CI) PPV**	**Confidence interval (95% CI) NPV**	**Accuracy**	**Fisher‘s exact test, *p*-value**
		**acute**	**long COVID**											
Lotta	Yes	10	4	73	0.9344	0.833	0.8432–0.9742	0.5520–0.9704	0.7143	0.9661	0.4535–0.8828	0.8846–0.994	0.9178	<0.0001
	No	2	57											
Baila	Yes	8	3	78	0.9545	0.6667	0.8747–0.9876	0.3906–0.8619	0.7273	0.9403	0.4344–0.9025	0.8563–0.9765	0.9103	<0.0001
	No	4	63											
Füge	Yes	10	6	61	0.8824	1	0.7662–0.9449	0.7225–1	0.625	1	0.3864–0.8152	0.9213–1	0.9016	<0.0001
	No	0	45											
Joe	Yes	10	0	86	1	0.8333	0.9507–1	0.5520–0.9704	1	0.9737	0.7225–1	0.9090–0.9953	0.9767	<0.0001
	No	2	74											
Vine	Yes	10	2	50	0.9487	0.9091	0.8311–0.9909	0.6226–0.9953	0.8333	0.9737	0.5520–0.9704	0.8651–0.9987	0.9400	<0.0001
	No	1	37											
Bella	Yes	10	0	68	1	1	0.9379–1	0.7225–1	1	1	0.7225–1	0.9379–1	1	<0.0001
	No	0	58											
Filou	Yes	10	2	58	0.9583	1	0.8602–0.9926	0.7225–1	0.8333	1	0.5520–0.9704	0.9229–1	0.9655	<0.0001
	No	0	46											
Erec	Yes	9	1	70	0.9825	0.6923	0.9071–0.9991	0.4237–0.8732	0.9	0.9333	0.5958–0.9949	0.8407–0.9738	0.9286	<0.0001
	No	4	56											
					**Mean Sp**	**Mean Se**	**95% CI of mean Sp**	**95% CI of mean Se**	**Mean PPV**	**Mean NPV**	**95% CI of mean PPV**	**95% CI of mean NPV**	**Mean accuracy**	**95% CI of mean accuracy**
					**0.9576**	**0.8668**	**0.9252-0.99**	**0.7539-0.9798**	**0.8292**	**0.9734**	**0.7158–0.9425**	**0.9513–0.9955**	**0.9426**	**0.9134–0.9717**

**Table 2:** Diagnostic performance of the scent detection dogs in test scenario IIa (Long COVID vs. negative controls).

**Dog**	**Detection**	**SARS-CoV-2** **disease status**		**Total number of presented samples**	**Diagnostic specificity (Sp)**	**Diagnostic sensitivity (Se)**	**Confidence interval (95% CI) Sp**	**Confidence interval (95% CI) Se**	**Positive predictive value (PPV)**	**Negative predictive value (NPV)**	**Confidence interval (95% CI) PPV**	**Confidence interval (95% CI) NPV**	**Accuracy**	**Fisher's exact test *p*-value**
		**Long COVID**	**negative**											
Bella	Yes	4	1	21	0.941	1.000	0.730–0.997	0.510–1.000	0.800	1.000	0.376–0.990	0.806–1.000	0.952	0.0008
	No	0	16											
Margo	Yes	5	1	23	0.941	0.833	0.730–0.997	0.437–0.992	0.833	0.941	0.437–0.992	0.730–0.997	0.913	0.001
	No	1	16											
Erec	Yes	4	0	19	1.000	1.000	0.796–1.000	0.510–1.000	1.000	1.000	0.510–1.000	0.796–1.000	1.000	0.0003
	No	0	15											
					**Mean Sp**	**Mean Se**	**95% CI of mean Sp**	**95% CI of mean Se**	**Mean PPV**	**Mean NPV**	**95% CI of mean PPV**	**95% CI of mean NPV**	**Mean accuracy**	**95% CI of mean accuracy**
					**0.961**	**0.944**	**0.876–1.000**	**0.705–1.000**	**0.878**	**0.980**	**0.612–1.000**	**0.896–1.000**	**0.955**	**0.847–1.000**

**Table 3:** Diagnostic performance of the scent detection dogs in test scenario IIb (Acute COVID-19 vs. negative controls).

**Dog**	**Detection**	**SARS-CoV-2** **infection status**		**Total number of presented samples**	**Diagnostic specificity (Sp)**	**Diagnostic sensitivity (Se)**	**Confidence interval (95% CI) Sp**	**Confidence interval (95% CI) Se**	**Positive predictive value (PPV)**	**Negative predictive value (NPV)**	**Confidence interval (95% CI) PPV**	**Confidence interval (95% CI) NPV**	**Accuracy**	**Fisher's exact test *p* value**
		**positive**	**negative**											
Bella	Yes	6	5	52	0.886	0.750	0.760–0.951	0.409–0.956	0.546	0.951	0.280–0.787	0.839–0.991	0.865	0.0005
	No	2	39											
Margo	Yes	4	2	24	0.900	1.000	0.699–0.982	0.510–1.000	0.667	1.000	0.300–0.941	0.824–1.000	0.917	0.0014
	No	0	18											
Erec	Yes	6	6	49	0.857	0.857	0.722–0.933	0.487–0.993	0.500	0.973	0.254–0.746	0.862–0.999	0.857	0.0004
	No	1	36											
					**Mean Sp**	**Mean Se**	**95% CI of mean Sp**	**95% CI of mean Se**	**Mean PPV**	**Mean NPV**	**95% CI of mean PPV**	**95% CI of mean NPV**	**Mean accuracy**	**95% CI of mean accuracy**
					**0.881**	**0.869**	**0.827–0.936**	**0.557–1.000**	**0.571**	**0.975**	**0.357–0.785**	**0.914–1.000**	**0.880**	**0.800–0.960**

**Figure 1:**
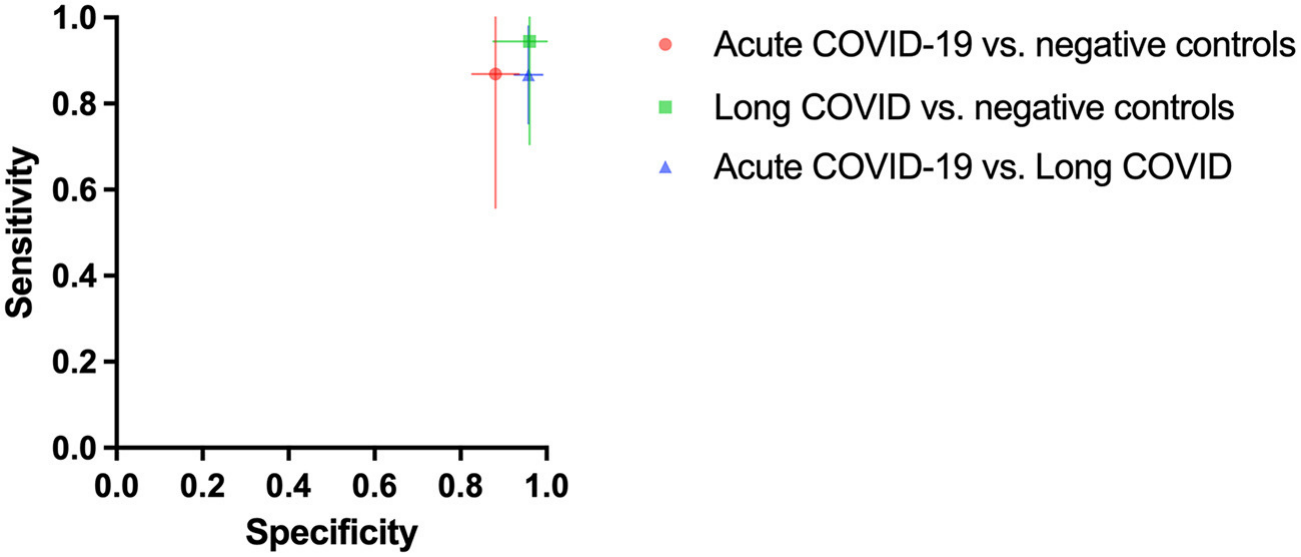
Mean diagnostic specificity and sensitivity for all dogs for acute COVID-19 vs. negative control (red circle), Long COVID vs. negative control (green square), and acute COVID-19 vs. Long COVID (blue triangle) samples, respectively. The 95% confidence intervals of the means for specificity and sensitivity are shown with horizontal and vertical bars, respectively.

As shown in Figure 1 dogs achieved a mean sensitivity of 86.7% (95%CI: 75.4–98.0%) and a specificity of 95.8% (95%CI: 92.5–99.0%) in test scenario I, where samples of acute COVID-19 vs. Long COVID were presented (Table 1). When dogs were confronted with Long COVID and negative control samples in scenario IIa, dogs achieved a mean sensitivity (for Long COVID) of 94.4% (95%CI: 70.5–100.0%) and a specificity of 96.1% (95%CI: 87.6–100.0%) (Table 2). In test scenario IIb, when acute SARS-CoV-2 positive samples and negative control samples were comparatively presented to the dogs, a mean sensitivity (for acute COVID-19) of 86.9% (95%CI: 55.7–100.0%) and a specificity of 88.1% (95%CI: 82.7–93.6%) could be attained (Table 3).

## Discussion

Several studies have shown that dogs can be trained to distinguish samples of acutely SARS-CoV-2-infected patients from samples of SARS-CoV-2-negative, healthy controls as well as from other viral infections with high diagnostic sensitivity and specificity (3–9). In the current study, trained SARS-CoV-2-detection dogs were confronted with samples of Long COVID patients for the first time. During their training period only samples of acutely SARS-CoV-2-infected patients were used as target scent.

92.86% of Long COVID samples were indicated as SARS-CoV-2-positive, when Long COVID samples were presented next to SARS-CoV-2-negative control samples. Interestingly, when Long COVID samples were presented next to acute SARS-CoV-2-positive samples, dogs only indicated 3.96% of Long COVID samples as positive. These results suggest that the disease-specific odor of acute COVID-19 is still present in the majority of Long COVID samples, but probably not to the same extent as in samples of acutely infected COVID-19 patients. In other words, when acute COVID-19 samples are presented next to Long COVID samples the dogs rather indicate the samples from acute cases, with the smell they were trained on. In a recently published study performed by Grandjean et al., dogs identified only 51.5% of Long COVID patients when they were presented next to healthy individuals (20). The lower percentage of identified Long COVID patients compared to our results (51.5 vs. 92.86%) might be explained by the differing sample quality as the samples used by Grandjean et al. were taken at home and were sent *via* mail without standardized freezing or cooling of the samples (20). Nevertheless, these results also support the hypothesis that the disease-specific odor of acute COVID-19 is still present in the majority of Long COVID samples, but probably not as strong as in samples of acutely infected COVID-19 patients (20).

The disease-specific odor that can be detected by dogs is thought to be determined by a specific pattern of VOCs. VOCs are produced by cell metabolism and released with breath, urine, saliva, blood, sweat and other body fluids (21). As viruses have no metabolism, the common hypothesis is that viruses change the metabolism of the infected host and therefore generate a special VOC pattern (22). The nature of these VOCs is currently being identified by several international laboratories in different countries and data suggest that SARS-CoV-2 infections create a specific VOC pattern (23–25).

Apart from detecting VOC patterns, dogs might also be able to directly detect viral proteins with their vomeronasal organ (VNO). The VNO can process a wide range of molecules, including proteins (26, 27). This fact and the results generated in the present study support data on persistence of SARS-CoV-2 as documented in the literature for post-COVID-19 condition patients (15–17). Up to date, it had not been demonstrated whether it corresponds to the replicative virus or not. The canine detection test supports the hypothesis that the virus still replicates at least to a limited extent, after the acute phase of COVID-19. It may be possible that this occurs in various body regions such as olfactory mucosa (15), brain (16), and in monocytes (17), even if a nasopharyngeal swab PCR has become negative.

The results of the current study could suggest the hypothesis of SARS-CoV-2 persistence in Long COVID patients months after their acute SARS-CoV-2 infection, but the study should be regarded as a pilot study due to inclusion of a limited number of patients. Further research with more patients and samples acquired from the same patient at different time points is needed, to evaluate to what extent the sensitivity of medical detection dogs may vary throughout the course of the infection. For a better understanding of the pathophysiology of post-COVID-19 condition, future studies with higher sample sizes should also address the questions if the nature of the symptoms influences the detection performance of the dogs, as there has been a variety of symptoms described for post-COVID-19 condition. Furthermore, studies characterizing disease specific VOCs, should generate a deeper understanding of what scent detection dogs detect in SARS-CoV-2 infected individuals.

## Data Availability Statement

The original contributions presented in the study are included in the article/Supplementary Material, further inquiries can be directed to the corresponding author/s.

## Ethics Statement

The studies involving human participants were reviewed and approved by Ethics Committee of Hannover Medical School (consent number 9042_BO_K_2020). The patients/participants provided their written informed consent to participate in this study. The animal study was reviewed and approved by German Armed Forces.

## Author Contributions

FT was responsible for the planning of the study, conducted and coordinated the sample acquisition, carried out data analyses, and drafted the manuscript. NtH, SM, and HV designed and coordinated the study, carried out the main practical work (NtH and PJ) and were responsible for data analyses (SM). AO and CS participated in the planning of the laboratory part of the study and were in charge for the legal permission for sample processing. CS carried out the laboratory work at the Research Center for Emerging Infections and Zoonoses including sample preparation, virus inactivation, and RT-qPCR. HE programmed the DDTS software and supported the dog training. IP, ND, and TW were in charge for the ethical approval, patient recruitment, and sample collection at Hannover Medical School. ES was involved in designing and coordinating the study and was responsible for the dog training and helped with data analyses. All authors have read and approved the final manuscript.

## Funding

The project was funded by the COVID-19 Research Network of the State of Lower Saxony (COFONI) through funding from the Ministry of Science and Culture of Lower Saxony in Germany (14-76403-184). This Open Access publication was funded by the Deutsche Forschungsgemeinschaft (DFG, German Research Foundation) within the programme LE 824/10-1 Open Access Publication Costs and University of Veterinary Medicine Hannover, Foundation.

## Conflict of Interest

HE was employed by KynoScience UG. The remaining authors declare that the research was conducted in the absence of any commercial or financial relationships that could be construed as a potential conflict of interest.

## Publisher's Note

All claims expressed in this article are solely those of the authors and do not necessarily represent those of their affiliated organizations, or those of the publisher, the editors and the reviewers. Any product that may be evaluated in this article, or claim that may be made by its manufacturer, is not guaranteed or endorsed by the publisher.
