# Supplementary material for: Detection of Post-COVID-19 Patients Using Medical Scent Detection Dogs—A Pilot Study
Source: Front Med (Lausanne). 2022 Jun 16;9:877259. doi: 10.3389/fmed.2022.877259 (PMC9245071; doi:10.3389/fmed.2022.877259)
Supplement: Supplementary file 1 [file Table_1.docx]

Supplemental Table 1. Characteristics of the samples used for the study

| **Sample ID** | **Sex** | **Age** | **SARS-CoV-2 RT-qPCR at acquisition** | **Symptom status of acute COVID-19 episode** | **Long COVID symptoms** | **Sample material** | **+/- BPL** |
| --- | --- | --- | --- | --- | --- | --- | --- |
| PS64 | m | 30 | positive | mild | N/A | saliva | + |
| PS21 | f | 43 | positive | mild | N/A | saliva | + |
| PS60 | m | 67 | positive | severe | N/A | saliva | + |
| PS35 | f | 76 | positive | asymptomatic | N/A | saliva | + |
| PS80 | f | 23 | positive | mild | N/A | saliva | + |
| PS79 | f | 56 | positive | mild | N/A | saliva | + |
| PS65 | f | 32 | positive | mild | N/A | sweat | + |
| PS60 | m | 67 | positive | severe | N/A | saliva | + |
| PS63 | f | 27 | positive | mild | N/A | urine | + |
| RE-2 | f | 41 | ND | severe | fatigue, dyspnea, concentration difficulties, hair loss, muscular weakness | saliva | + |
| RE-4 | f | 56 | ND | severe | fatigue, sleep disorder, concentration difficulties, headache | saliva | + |
| RE-6 | f | 34 | ND | mild | fatigue, concentration difficulties | saliva | + |
| RE-8 | m | 19 | ND | severe (ICU) | no symptoms at sample acquisition | saliva | + |
| RE-3 | f | 32 | ND | severe (ICU) | fatigue, concentration difficulties, oblivion, hair loss | saliva | + |
| RE-5 | f | 45 | ND | mild | fatigue, limb pain, concentration difficulties, memory deficits, headache, dyspnea | saliva | + |
| RE-9 | m | 18 | ND | severe (ICU) | sleep disorder, hyposmia, hypogeusia | saliva | + |
| RE-11 | f | 74 | ND | severe | exercise-induced dyspnea, concentration difficulties, fatigue | saliva | + |
| RE-12 | f | 45 | ND | mild | irritative cough, visual impairment | saliva | + |
| 189757 | f | 25 | negative | N/A | N/A | sweat | + |
| 189754 | f | 28 | negative | N/A | N/A | urine | + |
| 189425 | f | 25 | negative | N/A | N/A | saliva | - |
| 189748 | f | 23 | negative | N/A | N/A | saliva | + |
| 189427 | f | 25 | negative | N/A | N/A | sweat | - |
| 189426 | f | 25 | negative | N/A | N/A | urine | - |
| 189758 | f | 22 | negative | N/A | N/A | saliva | + |

f: female. m: male. N/A: not applicable. ND: not determined.
